# A large seroprevalence survey of brucellosis in cattle herds under diverse production systems in northern Nigeria

**DOI:** 10.1186/1746-6148-8-144

**Published:** 2012-08-25

**Authors:** Hassan M Mai, Peter C Irons, Junaidu Kabir, Peter N Thompson

**Affiliations:** 1 Department of Production Animal Studies, Faculty of Veterinary Science, University of Pretoria, Private Bag X04, Onderstepoort, 0110, South Africa; 2 Animal Production Programme, School of Agriculture and Agricultural Technology, Abubakar Tafawa Balewa University, P. M. B. 0248, Bauchi, Nigeria; 3 Department of Veterinary Public Health and Preventive Medicine, Ahmadu Bello University, Zaria, Nigeria

**Keywords:** Brucellosis, c-ELISA, Management systems, Northern Nigeria, RBPT, Seroprevalence

## Abstract

**Background:**

This study was carried out to investigate the status of brucellosis in cattle under various management systems in Adamawa, Kaduna and Kano states, northern Nigeria. Using multi-stage sampling, serum samples of 4,745 cattle from 271 herds were tested using the Rose-Bengal plate-agglutination test (RBPT) and positives were confirmed using a competitive enzyme-linked immunosorbent assay (c-ELISA).

**Results:**

Prevalence estimates were calculated by adjusting for sampling weights and where possible for test sensitivity and specificity. Thirty-seven percent of all animals were RBPT positive, and after confirmation with c-ELISA the overall animal-level prevalence, adjusted for sampling weights, was 26.3% (95% CI, 22.1%-31.0%). Of the herds sampled, 210 (77.5%; 95% CI, 68.6%-84.5%) had at least one animal positive to both tests; this did not differ significantly between states (*P* = 0.538). Mean within-herd seroprevalence in positive herds was 30.2% (95% CI, 25.3%-35.1%) and ranged from 3.1% to 85.7%. Overall animal-level seroprevalences of 29.2% (95% CI, 22.5%-36.9%) n = 1,827, 23.3% (95% CI, 18.9%-28.3%) n = 1,870 and 26.7% (95% CI, 18.8%-36.7%) n = 1,048 were observed in Adamawa, Kaduna and Kano states, respectively (*P* = 0.496). A significantly higher seroprevalence was found in males (38.2%; 95% CI, 31.7%-45.2%) than in females (24.7%; 95% CI, 20.4%-29.5%) (*P* < 0.001) and in non-pregnant females (27.8%; 95% CI, 22.9%-33.5%) than in pregnant females (17.2%; 95% CI, 13.6%-21.5%) (*P* < 0.001). Seroprevalence increased with increasing age (*P* < 0.001), from 13.5% (95% CI, 8.9%-19.9%) in cattle <4 years to 35.0% (95% CI, 28.5%-42.3%) in cattle >7 years. Seroprevalence also varied between management systems (*P* < 0.001): pastoral systems 45.1% (95% CI, 38.6%-51.9%), zero-grazing systems 23.8% (95% CI, 6.8%-59.2%), agro-pastoral systems 22.0% (95% CI, 17.3%-27.8%), and commercial farms 15.9% (95% CI, 9.5%-25.5%). Seroprevalence did not differ significantly between breeds or lactation status.

**Conclusion:**

This is the first large study to assess the prevalence of bovine brucellosis over a wide geographic area of northern Nigeria, in a variety of management systems and using accurate tests. The seroprevalence of brucellosis was high, and higher than results of previous studies in northern Nigeria. The pastoral management systems of the traditional Fulanis may be encouraging the dissemination of the disease. Public enlightenment of the farmers about the disease, vaccination and appropriate national control measures are recommended.

## Background

Brucellosis is one of the most important and widespread zoonoses in the world [1]. *Brucella abortus* infection in cattle is endemic in Nigeria, resulting in huge economic losses due to decreased calving percentage, delayed calving, culling for infertility, cost of treatment, decreased milk production, abortions, stillbirth, birth of weak calves and loss of man-hours in infected people [2-4]. Infection in bulls also causes orchitis, epididymitis, seminal vesiculitis and hygroma [5,6]. In Nigeria and some countries where cattle are kept in close association with sheep and goats, infection can also be caused by *B. melitensis*[3,7].

Infection occurs via contaminated feed or water, by inhalation, through the conjunctiva, or by contact with infected aborted materials; while calves become infected *in utero* or via infected colostrum or milk [8]. Venereal transmission has also been reported [9]. In fully susceptible herds, abortion rates vary from 30 to 70% [5]. Infection may be lifelong, and during subsequent pregnancies there is invasion of the gravid uterus and allantochorion; abortion rarely recurs, but uterine and mammary infection recurs [10]. Since the reproductive performance of these carrier animals is unaffected, they are retained in herds in Nigeria despite the presence of pathognomonic clinical signs in some cases, making effective control programmes extremely difficult.

Prevalence of bovine brucellosis varies widely across Nigeria, and between herds in the same area [11,12], with reported seroprevalences of 0.2% to 80.0% [13,14]. In institutional farms, abattoir surveys and other ranches or dairy farms in southern Nigeria, prevalence in cattle ranged between 3.7% and 48.8% [15-18], while in the traditional nomadic Fulani cattle herds, the prevalence was between 0.4% and 26% [3,11,12,19]. Recently, a within-herd prevalence of 32.2% on a prison cattle farm [20]; and 19.5% seropositive and 25.3% positive milk samples [21] were reported in northern Nigeria. Prevalence studies in other surrounding countries indicated 8.4% in Cameroon [22], 7% in Chad [23], 41% in Togo [24] and 6.6% in Ghana [25]. Brucellosis has also been reported in many other parts of Africa [26-32], although detailed information on its prevalence is still lacking for most countries [2].

Although prevalence is high and variable in many countries, surveillance for the disease is generally poor [2,3,14,33]. Factors assumed to be responsible for variation in prevalence include purchase of infected cattle from the market for replacement or upgrading, nature of animal production, demographic factors, regulatory issues, climate, deforestation and wildlife interaction [19,26,33-35]. Furthermore, one major factor contributing to the spread of the disease is the free movement of animals practiced by the nomadic Fulani herdsmen, who own about 95% of all food animal populations in Nigeria [3,19,36]. Other factors that may influence the prevalence of brucellosis in Nigeria include management system [11,37], the herding of different species together [3,7,20], use of common pastures and water sources [14], age [12,18,21], breed [17,18], sex [21,37], lactation status [21] and season [11,14]. However, other variables such as pregnancy status and state have not been assessed. All these risk factors need to be taken into consideration in designing and execution of effective control programmes in Nigeria.

In the development of a livestock industry, disease eradication and control are paramount. The continuous movement of cattle as a result of trade and for grazing is a common practice in Nigeria, putting many herds at risk of brucellosis infection. So too do the sharing of bulls and use of open-range grazing. Reports have indicated that trade cattle in and from the northern states, and also those from across the northern borders of Chad and Niger showed evidence of infection [14,17,18]. More than 20% of the trade cattle came from outside Nigeria [17]. The nature of the communal grazing systems used by the nomadic Fulanis, as well as porosity of the borders, predisposes livestock in the study area to infection, with a serious risk to human health [11,17,36]. Even in developed countries, despite the preventive and control measures that exist; there is still a high potential for transmission and spread of *Brucella* organisms via animals and their products [38]. Recent estimates of losses in meat and milk production as a result of brucellosis are $800 million annually in the USA [39], in excess of $224 million in Nigeria [16] and $37.5 million in South Africa [40].

Many of the studies conducted on brucellosis in Nigeria have been from the humid southern part [15-18] and were mainly abattoir surveys which were not representative of the general population. Abortions in Adamawa Province in the late 1940’s caused so much concern that *B. abortus* strain 19 vaccination was implemented in 1949, 1953 and 1956 [41]. Since then no surveys to our knowledge have been done on brucellosis in Adamawa Province, except that of Atsanda and Agbede [37] who used the Rose-Bengal plate agglutination test (RBPT) and serum agglutination test (SAT), and very recently Bertu *et al.*[14] who analysed some samples collected from sick animals using RBPT and a rapid field test. Most studies in northern Nigeria were based on small sample sizes or suspect samples submitted to laboratories, or were done in restricted locations or in abattoirs [3,4,12-14,19-21]. In addition, most studies in Nigeria have relied on the relatively non-specific RBPT and few have used a more specific confirmatory test such as enzyme-linked immunosorbent assay (ELISA) [12]. Reported sensitivity and specificity of RBPT in Zambian cattle were 90.0% and 75.0% [42] and for c-ELISA they were 98.0% and 99.0%, respectively [43].

This study was prompted by an apparent increase in the occurrence of bovine brucellosis in Nigeria [3,21], and therefore the need to obtain an accurate estimate of its prevalence and examine the role of the commonly practiced traditional management system of pastoral Fulanis. There is a dearth of studies using a specific diagnostic test and covering wider geographical areas and different management systems. The objective of this study therefore was to use a structured, multistage sampling strategy, combined with a sensitive and specific diagnostic test, to estimate the animal- and herd-level seroprevalence of bovine brucellosis in three states of northern Nigeria. A secondary objective was to estimate and compare seroprevalence between different management systems and to assess the effect of certain animal-level risk factors on seropositivity in both private and government herds, including some settled and pastoral Fulani herds that usually resist attempts to evaluate their herds.

## Methods

The research protocol for this study was approved by the Animal Use and Care Committee of the University of Pretoria (Protocol no. V073-08).

### Selection of study states

**Figure 1:**
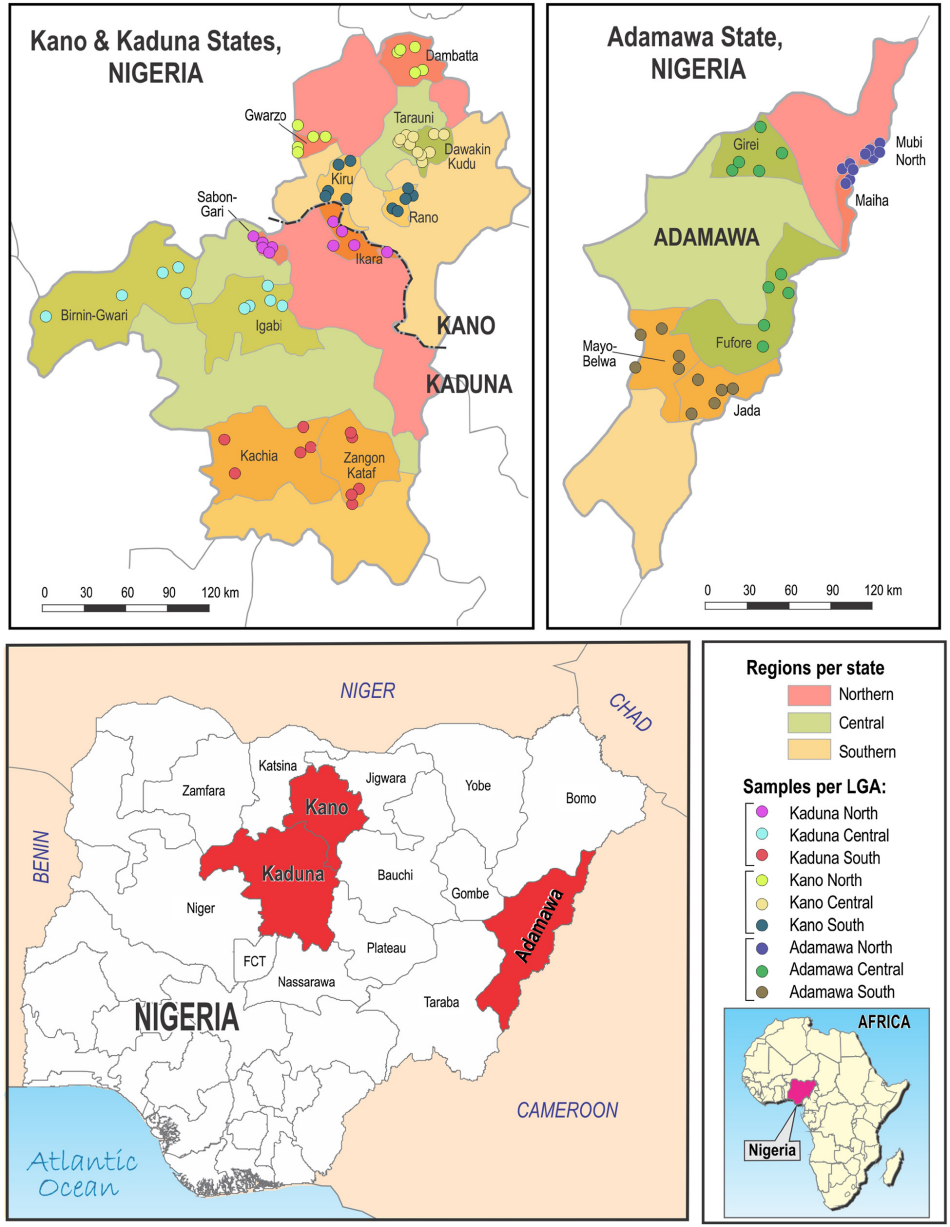
Map of Nigeria showing 3 states, 18 local government areas and 89 wards sampled.

Three states out of the nineteen were sampled from the northern region of Nigeria. The states selected were Kaduna, Kano and Adamawa (Figure 1). Their selection was based on their location, proximity to a reliable laboratory, farming systems, human and cattle populations, cooperation from farmers, sharing of international borders and variety of cattle breeds.

### Adamawa state

The state has a total land area of 42,159 km^2^ and a cattle population of 3.8 million, lying between latitudes 8°N and 11°N and longitudes 11.5°E and 13.5°E [44]. There are two main vegetation zones in the state: the sub-Sudan characterized by short grasses and short trees commonly found in the northern parts and the Guinea savannah zones where the vegetation is thick with tall grasses and trees in the southern parts. Average daily temperature between 15.2°C and 42°C, and relative humidity ranging from 27-79% were recorded. The rainy season commences from May and ends in October, with average annual rainfall of 1600 mm in the southern parts and 759 mm in the northern parts [45].

### Kaduna state

Kaduna state has a land area of 48,473 km^2^ and a cattle population of 3.1 million, and is located between latitudes 9°N and 11.3°N and longitudes 10.3°E and 9.6°E [44]. The state extends from the Sudan savannah in the north to the tropical grassland of the Guinea savannah in the south. Daily temperatures range from 14-30°C with a relative humidity of 12-72%. The rainy season is usually from April through November, with greater variation in the northern part. The annual rainfall varies, decreasing from an average of about 1530 mm in the southeast to about 1015 mm in the northeast [46,47].

### Kano state

This state has a land area of 42,593 km^2^ and cattle population of 3.2 million. It is situated at latitudes 12°N and longitudes 9°E [44]. The location is within the Sudan savannah in the north and the Guinea savannah vegetations in the south which provides ample natural fodder for cattle to graze. The temperatures range from 26-40°C with a relative humidity of 11-68%. The rainfall with a duration of about 3–5 months between May and September, ranges from over 1,000 mm in the extreme south to a little less than 800 mm in the extreme north [48,49].

### Sample size

To calculate the required number of farms to be sampled in order to estimate the prevalence of *B. abortus*-infected herds*,* an expected herd prevalence (*P*_*exp*_) of 40%, desired absolute precision (*d*) of 10% and a confidence level of 95% were applied using the formula *n* = 1.96^2^*P*_exp_ (1 – *P*_exp_)/d^2^[50], resulting in required sample size of 93 farms. However, multistage cluster sampling was used because of its practical advantages and flexibility. Therefore, the design effect (*D*) of the survey was calculated using the formula *D* = 1 + (*b* – 1) *roh*[51], where *b* is the average number of samples per cluster and *roh* is the rate of homogeneity, equivalent to the intra-cluster correlation coefficient (*ρ*) in single-stage cluster sampling. It was decided to sample approximately 12 to 13 farms per local government area (*b* = 13). An intra-cluster correlation coefficient of *ρ* = 0.09 was reported for *B. abortus* in cattle [52]; in order to account for the multistage design, a higher value of 0.15 was used for *roh*. The design effect was therefore calculated to be *D* = 2.8 which, multiplied by the original calculated sample size, gave a required sample size of 261 farms. Ultimately, a total of 271 herds was sampled.

### Survey design

Each of the three selected states was divided into three geographic zones: northern, central and southern (Figure 1). Six local government areas (LGA) were randomly selected from each state (2 LGA per geographic zone), using as sampling frame a list of all LGA in each zone. Similarly, within each selected LGA, approximately 50% of the wards (4 to 6 per LGA) were randomly selected (Figure 1). Within each selected ward, only available herds with at least ten mature females were used. Since no sampling frames were available for selection of herds within wards, herds were selected by visiting the farms and taking the first few that agreed. An average of three herds was selected per ward, giving an average of 15 herds selected per LGA. Animals sampled in each selected herd included all the breeding bulls and other mature bulls, first calf heifers that had calved at least six weeks previously, and all the mature cows.

The following four management systems were encountered: 1) a pastoral system which is practiced by the Fulanis that move for long distances from location to location in search of pasture during the dry season, while in the rainy season their animals graze close by since pasture is available; 2) an agro-pastoral system in which animals do not travel long distances, but graze communally and return in the evening, and are given supplementary feeds, including crop residues, particularly during the critical period of the dry season; 3) commercial farms that are intensively managed, usually fenced and in some cases paddocked, and where the cattle are well supplemented with feeds in addition to sown and natural pastures; and 4) zero-grazing herds that are also intensively managed but the cattle are more restricted or tethered in one location and are provided with feeds where they are confined.

### Demographic data and sample collection

Blood was collected from the jugular, coccygeal or saphenous veins into Vacutainer® tubes, which were immediately placed into an ice bath and transported to the laboratory within a maximum of 7 hours. When the outside ambient temperature was cool, the clot was allowed to form in the vacutainer tube in the field before transportation. The samples were centrifuged at 3,000 rpm for 15 minutes and the serum was removed and stored at −20°C until analyzed.

Pre-tested and structured questionnaires were administered during the sample collection to determine the profile of the animal, including the presence of hygroma, orchitis or epididymitis, as well as information about the farm and herd. Pregnancy diagnosis was determined by rectal palpation. Age was estimated using farm records, dentition and, in some cases, cornual rings.

Data obtained from the serological test and questionnaires were stored in a spreadsheet programme (Excel 2007; Microsoft Corp., Redmond, WA, U.S.A.).

### Screening using Rose-Bengal plate-agglutination test

The RBPT (VLA, Weybridge, UK) was done on all samples in accordance with the manufacturer’s instructions. All samples testing positive or which were inconclusive using the RBPT were further subjected to c-ELISA.

### Confirmation using competitive enzyme-linked immunosorbent assay

This test was performed using a competitive ELISA (c-ELISA) kit (COMPELISA, VLA, Weybridge, UK) according to the manufacturer’s instructions, in order to confirm the RBPT positive and inconclusive samples. The optical density (OD) was measured at 450 nm using a microplate ELISA reader (SIGMA DIAGNOSTICS EIA Multi-well Reader II). A positive/negative cut-off was calculated as 60% of the mean OD of the four conjugate control wells. Any test sample giving an OD equal to or below this value was regarded as positive.

### Data analysis

A positive herd was defined as any herd that had at least one animal positive to both RBPT and c-ELISA. For each ward, the sampling fraction was calculated as the product of the proportion of wards sampled within each LGA and the proportion of LGAs sampled within each state. The sampling weight was then calculated as the inverse of the sampling fraction. Because it was not possible to calculate the proportion of farms sampled within each ward, and because all eligible animals on each farm were tested, the same sampling weight was assigned to every animal within a ward. Estimates of the animal- and herd-level seroprevalences were calculated by state, management system, age, sex and breed using the ‘*svy*’ command in STATA 12, which accounts for sampling weights, stratification and clustering in the multistage survey design to produce adjusted prevalence estimates and standard errors. Seroprevalence estimates were then compared using the Chi-square test, corrected for the survey design using the second-order correction of Rao and Scott [53]. Animal-level prevalences were also calculated and were adjusted for the sensitivity and specificity of the serial testing system. The sensitivity of the test series was calculated as: *Se* = *Se*_RBPT_ × *Se*_ELISA_ = 90.0% × 98.0% = 87.9% and the specificity was calculated as *Sp* = 1 – (1 – *Sp*_RBPT_) × (1 – *Sp*_ELISA_) = 1 – (1 – 75.0%) × (1 – 99.0%) = 99.8%. True prevalence was then calculated using the formula of Rogan and Gladen [54]: *TP* = (*AP* + *Sp* – 1) / (*Se* + *Sp* – 1), where *AP* = apparent prevalence, *Se* = sensitivity of the test series, *Sp* = specificity of the test series.

To adjust for confounding amongst animal-level factors (age, sex and breed), as well as state and management system, their association with brucellosis seropositivity was assessed using a hierarchical mixed-effects logistic regression model. Age, sex, breed, state and management system were included as categorical fixed effects. LGA, ward and herd were included as nested random effects, with ward nested within LGA and herd nested within ward. In addition, data were restricted to females only and pregnancy and lactation status were added to the model in order to estimate their association with brucellosis seropositivity. No variable selection or elimination was done. Fit of the models excluding the random effects was assessed using the Hosmer-Lemeshow goodness-of-fit test. All analyses were done using STATA 12 (Stata Corporation, College Station, TX, USA) and a significance level of 5% was used.

## Results

Data from 4,745 samples from 271 herds were available for analysis. Of the 271 herds included in the study, there were 225 herds (84.9%) with at least one animal testing positive based on RBPT and 210 herds (77.5%) based on c-ELISA. The herd-level seroprevalence of brucellosis in the three states combined, adjusted for the sampling weights, was estimated to be 77.5% (95% CI, 68.6%-84.5%). The herd-level seroprevalences for both tests in the individual states adjusted for the sampling weights are shown in Table 1. Twenty three per cent of all herds sampled and 30% of the infected herds had animals with hygromas (Table 1). Of the 63 herds in which hygromas were present, 54 (64.3%) were from Adamawa, 7 (13.7%) from Kano and 2 (2.7%) from Kaduna states (Table 1). Only one herd with animals with hygromas (1.6%) was negative for brucellosis.

**Table 1:** Herd-level seroprevalence of bovine brucellosis based on RBPT and c-ELISA, adjusted for sampling weights, and presence of hygroma, orchitis or epididymitis in three states of northern Nigeria

**State**	**n**	**RBPT pos. (%)**	**c-ELISA pos. (%)**	**95% CI**	**Hygroma (%**^**#**^**)**	**Orchitis/ Epididymitis (%**^**#**^**)**
Adamawa	100	89 (88.0)	84 (82.3)	66.8 - 91.5	54 (64.3)	12 (14.3)
Kaduna	105	78 (74.8)	75 (72.0)	58.6 - 82.4	2 (2.7)	12 (16.0)
Kano	66	58 (89.6)	51 (78.5)	61.2 - 89.4	7 (13.7)	7 (13.7)
Overall	271	225 (84.9)	210 (77.5)	68.6 - 84.5	63 (30.0)	31 (14.8)

Key:

n: Sample size.

c-ELISA: Competitive enzyme-linked immunosorbent assay.

RBPT: Rose-Bengal plate-agglutination test.

CI: Confidence Interval.

^#^ Percentage of c-ELISA-positive herds showing hygroma, orchitis or epididymitis.

A total of 4,745 samples was tested with RBPT, of which 1,735 (36.6%) were positive for brucellosis. Of these, 1,137 (65.5%) were confirmed to be seropositive for brucellosis upon further testing by c-ELISA, giving 34.5% overall false positives (42.8%, 24.7% and 27.1% in Adamawa, Kaduna and Kano states, respectively) (Table 2). Based on c-ELISA, the estimated overall survey adjusted true animal-level seroprevalence was 26.3% (95% CI, 22.1%-31.0%) (Table 3). The prevalence for Adamawa state was 29.2% (95% CI, 22.5%-36.9%), for Kaduna state 23.3% (95% CI, 18.9%-28.3%) and for Kano state 26.7% (95% CI, 18.8%-36.7%) (Table 3).

**Table 2:** Animal-level seroprevalence of bovine brucellosis in three states of northern Nigeria based on RBPT and c-ELISA tests

**State**	**n**	**RBPT pos. (%)**	**c-ELISA pos. (%)**	**FP (%)**
Adamawa	1827	892 (48.8)	510 (27.9)	382 (42.8)
Kaduna	1870	511 (27.3)	385 (20.6)	126 (24.7)
Kano	1048	332 (31.7)	242 (23.1)	90 (27.1)
Overall	4745	1735 (36.6)	1137 (24.0)	598 (34.5)

Key:

n: Sample size.

RBPT: Rose-Bengal plate-agglutination test.

c-ELISA: Competitive enzyme-linked immunosorbent assay.

FP (%): False positives (proportion of animals that were RBPT positive but c-ELISA negative).

**Table 3:** Animal-level seroprevalence of brucellosis in cattle in three states of northern Nigeria, by breed, sex, age, management system, pregnancy status and lactation status, adjusted for sampling weights and test sensitivity and specificity

**Variable and level**	**n**	**Adjusted prev. (%)**	**95% CI (%)**	*P*-value*
**All Animals**				
State				0.496
Adamawa	1827	29.2	22.5-36.9	
Kaduna	1870	23.3	18.9-28.3	
Kano	1048	26.7	18.8-36.7	
Management system				<0.001
Zero-grazing	101	23.8^ab^	6.8-59.2	
Commercial	642	15.9^a^	9.5-25.5	
Agro-pastoral	2758	22.0^a^	17.3-27.8	
Pastoral	1244	45.1^b^	38.6-51.9	
Breed				0.392
Bunaji	3052	27.5	22.5-33.2	
Gudali	863	26.3	22.1-31.1	
*Bos taurus*	118	15.1	6.6-31.0	
*B. taurus x B. indicus*	267	21.8	11.7-37.0	
Other *B. indicus*	445	24.7	17.8-33.5	
Sex				<0.001
Males	596	38.2^a^	31.7-45.2	
Females	4149	24.7^b^	20.4-29.5	
Age				<0.001
< 4 years	559	13.5^a^	8.9-19.9	
4 - 5 years	2038	23.8^b^	19.9-28.1	
5 - 7 years	1312	29.8^c^	22.6-38.2	
> 7 years	804	35.0^c^	28.5-42.3	
**Females Only**				
Pregnancy status				<0.001
Pregnant	1367	17.2^a^	13.6-21.5	
Non-pregnant	2835	27.8^b^	22.9-33.5	
Lactation status				0.635
Lactating	1819	25.3	20.7-30.5	
Non-lactating	2386	23.2	19.3-29.9	
**Total**	4745	26.3	22.1-31.0	

Figures with different superscripts within the same variable differ significantly (*P* < 0.05).

* *P*-value determined using the Chi-square test, corrected for the survey design using the second-order correction of Rao and Scott [53].

**Figure 2:**
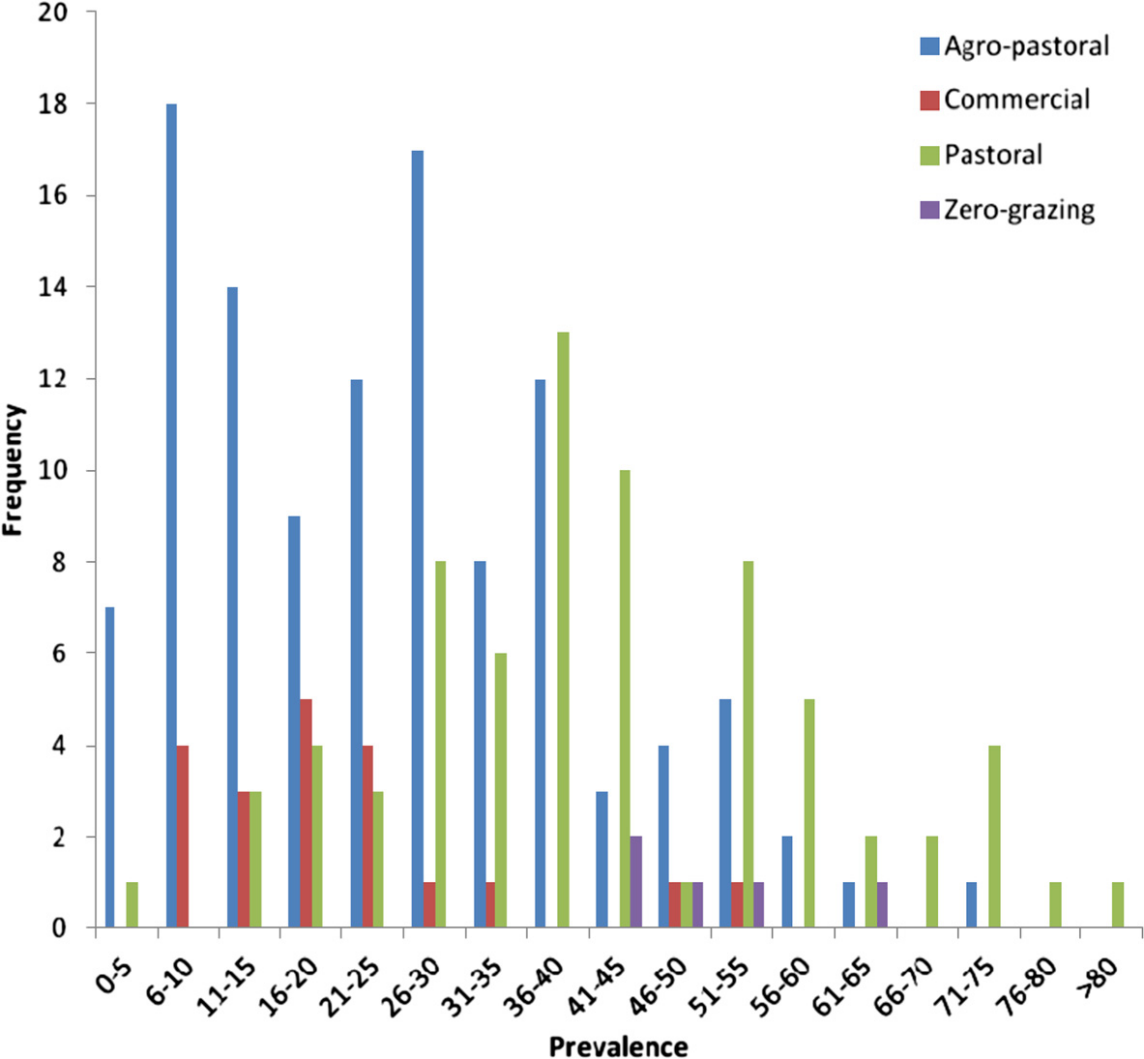
Distribution of within-herd prevalence of seropositive animals in brucellosis-positive herds in the different management systems.

Overall mean within-herd seroprevalence of brucellosis was 24.6%. Within positive herds, the mean prevalence of seropositive animals, adjusted for survey design, was 30.2% (95% CI, 25.3%-35.1%) and ranged from 3.1% to 85.7%. The distribution of within-herd seroprevalence in infected herds for the various management systems is shown in Figure 2. Mean within herd seroprevalences in the different management systems, from lowest to highest, were: commercial 18.5% (95% CI, 10.2%-26.8%), agro-pastoral 25.2% (95% CI, 19.0%-31.4%), pastoral 43.0% (95% CI, 38.6%-47.5%) and zero-grazing 51.1% (95% CI, 46.9%-55.3%).

The seroprevalence differed significantly between males (38.2%; 95% CI, 31.7%-45.2%) and females (24.7%; 95% CI, 20.4%-29.5%) (*P* < 0.001), between cattle <4 years (13.5%; 95% CI, 8.9%-19.9%) and >7 years of age (35.0%; 95% CI, 28.5%-42.3%) (*P* < 0.001), between commercial farms (15.9%; 95% CI, 9.5%-25.5%) and pastoral farms (45.1%; 95% CI, 38.6%-51.9%) (*P* < 0.001), and between non-pregnant (27.8%; 95% CI, 22.9%-33.5%) and pregnant females (17.2%; 95% CI, 13.6%-21.5%) (*P* < 0.001). There was no significant difference in seroprevalence between lactating (25.3%; 95% CI, 20.7%-30.5%) and non-lactating (23.2%; 95% CI, 19.3%-29.9%) animals (*P* = 0.635) or between breeds (*P* = 0.392), although *Bos taurus* had the lowest prevalence of 15.1% (95% CI, 6.6%-31.0%) and Bunaji had the highest prevalence of 27.5% (95% CI, 22.5%-33.2%) (Table 3).

The associations between animal-level factors and brucellosis seropositivity, adjusted in the multivariable model for confounding both by the other animal-level factors and by state and management system, are shown in Table 4. The greater odds of seropositivity in males (OR = 1.98; 95% CI, 1.54-2.54; *P* < 0.001) remained, as did the monotonic increase with increasing age (e.g., for >7 y vs. < 4 y: OR = 3.82; 95% CI, 2.72-5.36; *P* < 0.001) and the increased odds in non-pregnant compared to pregnant cows (OR = 1.84; 95% CI, 1.49-2.27; *P* < 0.001). Neither state nor management system acted as confounders of the effects of the above variables, as their exclusion resulted in <10% change to coefficients. Although there was ultimately no significant effect of breed, there was some confounding both by state and by management system, with their exclusion from the model resulting in up to 66% and 180% change in coefficients, respectively. Similarly, there was some confounding of the effect of lactation status on seropositivity by both state and management system. The odds of seropositivity were significantly higher in the pastoral management system than in the other management systems (e.g., vs. agro-pastoral: OR = 3.52; 95% CI, 2.50-4.95; *P* < 0.001). The random effects in the hierarchical model showed that there was significant variation in brucellosis seropositivity between herds within ward and between wards within LGA, but not between LGAs within state.

**Table 4:** Animal-level risk factors for brucellosis seropositivity, adjusted for state and management system: results from hierarchical mixed-effects logistic regression models

**Risk factor and level**	***OR***	***95% CI (OR)***	***P-value***^***#***^
**All Animals**			
Breed			
*Bos taurus*	1*	–	–
Gudali	1.52	0.65 - 3.54	0.334
Bunaji	1.52	0.66 - 3.50	0.327
Other *B. indicus*	1.59	0.67 - 3.75	0.293
*B. taurus* x *B. indicus*	1.71	0.71 - 4.11	0.230
Sex			
Females	1	–	–
Males	1.98	1.54 - 2.54	<0.001
Age			
< 4 years	1	–	–
4 - 5 years	1.70	1.26 - 2.31	0.001
5 - 7 years	2.50	1.82 - 3.44	<0.001
> 7 years	3.82	2.72 - 5.36	<0.001
**Females Only**			
Pregnancy status			
Pregnant	1	–	–
Non-pregnant	1.84	1.49 - 2.27	<0.001
Lactation status			
Lactating	1	–	–
Non-lactating	1.17	0.96 - 1.43	0.112
**Confounders**			
State			
Kaduna	1	–	–
Kano	1.09	0.66 - 1.78	0.737
Adamawa	1.54	0.96 - 2.45	0.073
Management system			
Agro-pastoral	1	–	–
Commercial	1.06	0.55 - 2.04	0.860
Zero-grazing	1.32	0.53 - 3.31	0.555
Pastoral	3.52	2.50 - 4.95	<0.001
**Random effects**			
LGA	–	–	1.000
Ward	–	–	<0.001
Herd	–	–	<0.001

^#^ Wald *P*-value.

* Reference level.

## Discussion

The study revealed that bovine brucellosis is still prevalent in the three states of northern Nigeria covered, with a herd-level prevalence of 77.5%, higher than the 40% reported in Zimbabwe [31], 42% in Ethiopia [28], 56% in Uganda [27] and 63% in Brazil [55]. Interestingly, a very similar herd prevalence of 77.8% was reported 40 years ago in southern Nigeria [15]. The dissemination of Ndama cattle, reportedly the most heavily infected breed [17], to various parts of the country as foundation stocks because of their good beef conformation and resistance to trypanosomosis and dermatophilosis infection may have contributed to the high prevalence in other parts of the country. Other interstate movement and trade in cattle across the country, as well as the nomadic nature of the pastoral Fulanis may also have contributed to high infection rates [14,17,18,36].

Livestock production in Nigeria was dominated by nomadic pastoralism long before the advent of colonial era. In the 1930s, the government established stock farms for dairy herds by selective breeding. In the same period, mixed farming policy hence agro-pastoral production system as well as range management were introduced for livestock improvement in Nigeria [56]. In the 1940s to 1950s, government investigation and breeding centres in settled herds all over the country and artificial insemination were established. It was also within this period that exotic breeds of cattle were introduced to upgrade the local stock [56]. Brucellosis infection rate of over 30% was reported during the 1940s at various livestock centres in Nigeria characterized by abortion storms [15,17]. Attempts were made to vaccinate cattle against brucellosis but it was limited and irregular [17,18]. Between 1970s and 1990s, about 96% of the cattle were zebu-type cattle, most of which were tended by traditional Fulani pastoralists [57]. In addition, in 1970s, 30 to 40% of the beef consumed in Nigeria was imported from Niger, Chad, and other neighboring countries [57]. These factors may have influenced the increase in prevalence of bovine brucellosis in Nigeria and elsewhere [3,38].

An overall adjusted animal-level true prevalence of 26.3% (95% CI, 22.1-31.0%) was obtained in this study (Table 3). Of the three states sampled, Adamawa state had the highest apparent animal-level seroprevalence of 29.2%, although this was not significantly higher than in Kano (26.7%) or Kaduna (23.3%). However, after adjustment for confounding, the difference between Adamawa and Kaduna approached significance (*P* = 0.07). In addition, Adamawa state showed the highest number of cattle exhibiting hygroma, seen in 54 of 84 positive herds (Table 1). Adamawa state borders on Cameroon, and constant trans-border movement of cattle has been reported to result in transmission of contagious bovine pleuropneumonia [58]. Cross-border movement has been implicated in the transmission of brucellosis by previous investigators in Nigeria [17,19] and elsewhere [25,59], and although not directly implicated by this study, it is possible that it may be a risk factor for brucellosis in Adamawa state.

The variations in the results of the two tests showed that many of the RBPT results were falsely positive because of its relatively low specificity, with Adamawa state showing the greatest discrepancy between the two tests (Table 2). The c-ELISA of all the samples was done in the same laboratory at the same time, while screening using RBPT was done in two different laboratories at different times but by the same investigator, thereby reducing the possibility of laboratory error or subjective interpretation. Since none of the herds sampled had been vaccinated against brucellosis, the antibodies responsible for the false positives were likely from other sources. Some bacterial pathogens such as *Yersinia enterolitica* serovar IX, *Vibrio cholera*, *Escherichia coli* O:157, *Salmonella* spp. and *Sternotrophomonas maltophilia* have been reported to produce cross reacting antibodies to brucellosis [60,61], with *Y. enterolitica* being the most significant cause of false positives. It is possible that the prevalence of one or more sources of cross-reacting antibodies was higher in Adamawa than in the other two states. In a Zambian study, for every three positive RBPT animals, only one tested positive on c-ELISA, except among animals which had aborted, where the ratio was close to one [30]. The c-ELISA test, with a higher specificity than RBPT, complement fixation test and florescent polarization assay, and therefore an ideal confirmatory test [42], has however rarely been used in Nigeria in naturally infected cattle, and never in a large study including different production systems.

The animal-level prevalence reported in this study (26.3%) is higher than recent reports from northern Nigeria [21]. Furthermore, the prevalence is much higher than the 9.8% and 18.6% using RBPT and MRT respectively in indigenous cattle in abattoirs in western Nigeria [18], 20% in government farms in the north using SAT [62], and 6.6% in cattle herds in northern Nigeria using ELISA [12], but lower than a recent report of 45% from samples of sick animals in Adamawa state, Nigeria, using RBPT and rapid field test [14]. However, the result is consistent with the 32% within-herd prevalence reported in one prison farm in northern Nigeria using SAT and MRT [20]; and the 38.0% using RBPT and SAT in cattle in government Livestock Investigation and Breeding Centers in Kano [19]. Studies elsewhere showed prevalences between 49% and 60% among breeding cows and heifers, dairy farms and abattoir surveys in the southern and western states of Nigeria on the basis of SAT, with CFT on doubtful results [15,17]. The animal-level prevalence obtained in this study was also much higher than those reported in South Africa [32], North Africa [63] and East Africa [28,64]. However, a higher prevalence of 41% was reported in Togo, West Africa [24]. McDermott and Arimi [2] also reported a higher prevalence in sub-Saharan Africa. Although some of the variation in results between studies may be due to the use of different diagnostic techniques, considering only those studies that used the same serological test as the present study, the prevalence of bovine brucellosis appears to be increasing in northern Nigeria. Several reports have previously indicated that brucellosis is on the increase in Nigeria [3,19,21] and other developing countries [2,33]. Lack of proper surveillance and control measures in most parts of Africa may be contributing to this increase, as may the importation of animals and their products from more developed countries despite the preventive and control measures in such countries [2,33,38]. Nevertheless, despite reports showing the extent of brucellosis in Nigeria, there is no record of a proper brucellosis control programme in the country [3,14].

Traditional Fulani herds practicing nomadism or pastoralism showed the highest prevalence followed by the zero-grazing and agro-pastoral systems, with the lowest prevalence being recorded in commercial herds. The findings by Nuru and Dennis [11], Bale and Kumi-Diaka [19], and Ocholi *et al*. [12] who reported prevalences of between 0.4% and 26% in traditional nomadic Fulani herds; and Atsanda and Agbede [37] who reported a slightly higher infection rate of 5.1% among nomadic cattle herds than among settled cattle herds (4.4%) in Adamawa state, are consistent with the findings of our study. Furthermore, Ocholi *et al*. [3] and Rikin [36] reported the prevalence of brucellosis to be rising among pastoral and semi-pastoral herds which comprise about 95% of the cattle population in Nigeria [11]; Bale and Kumi-Diaka [19] indicated free movement of the pastoral Fulani herdsmen and interaction of cattle with those of other Fulani herdsmen as major factors in spreading brucellosis. These observations agree with our findings, as do reports by McDermott and Arimi [2] and Matope *et al*. [31] of highest occurrence in pastoral production systems in arid and semi-arid areas in Zimbabwe and other parts of Africa, and Bernard *et al*. [27] in Uganda and Berhe *et al.*[28] in Ethiopia who also reported a higher seroprevalence in the transhumance system than in sedentary cattle.

The odds of brucellosis seropositivity were 3.5 times greater amongst pastoral herds than agro-pastoral herds in this study. The high prevalence of brucellosis in a pastoral management system may partly be attributed to long distance movement of cattle in search of pasture and water and co-mingling in communal grazing areas and at watering points, particularly during the dry season. Musa *et al*. [26] observed in Sudan that clinical manifestation of brucellosis often began during adverse weather conditions and famine. During such times animals become concentrated on scarce pastures and around watering points, which may become contaminated with aborted foetal materials or fluids from infected normal calvings. Many pastoralists do not isolate cows during parturition or dispose of the placenta following calving, resulting in contamination of the environment and transmission of brucellosis within and between herds. Other possible risk factors for brucellosis associated with the pastoral management system in Nigeria include bull sharing which may result in venereal transmission of brucellosis [9], purchasing of livestock from markets without quarantine [19] and interaction of cattle with wildlife [34,35].

The prevalence of brucellosis in zero-grazing systems in this study was also high. This is contrary to the report by McDermott and Arimi [2] of low prevalence due to very low level of between-herd contacts. However, Bayemi *et al.*[22] and Karimuribo *et al.*[29] observed a high prevalence in intensively managed herds. Cattle in zero-grazing systems in Nigeria are generally bought from the open market for a fattening programme, which may explain the high prevalence in such systems.

Reports indicate that about 20% of infected pregnant animals do not abort, while 80% of animals that abort as a result of *B. abortus* infection, do so only once [65] and thereafter will usually carry the pregnancy to full-term and appear healthy. In herds that have chronic brucellosis and do not introduce new animals, very few or no abortions occur and the disease is almost impossible to recognise clinically [66]. The emphasis in livestock production in Nigeria is on the ability of the females to produce calves; as long as cows produce, farmers tend to keep them, even if they have a history of abortion. In bulls, brucellosis causes no impairment of libido or breeding capacity [6] and the disease is subclinical in most animals [5]. For these reasons, farmers seldom cull infected animals from their herds, contributing to the high prevalence observed in this study. These apparently healthy cattle that are reproducing normally serve as permanent carriers of brucellosis. Some cattle may get rid of the infection within a few months, while others may remain infected for life, thereby transmitting the disease at subsequent parturitions [8,65]. This scenario could make control of the disease in Nigeria an extremely difficult task, requiring a well-designed and coordinated eradication policy and good cooperation of all sectors of the industry. Strategies such as immunization and the identification of and selection for genetic resistance factors may be required to make significant progress in control of the disease.

Cows with visible hygromas, but reproducing normally are also left in the herds. All forms of hygromas were encountered in this study including fluid accumulation in some infected animals on the cervical region, between the nuchal ligament, shoulder, flank, primary thoracic spines and most commonly the carpal and stifle joints. An additional picture file shows this in more detail (see Additional file 1). Over 23% of all herds sampled and 30% of the infected herds had hygroma (Table 1). This is the first report of this manifestation of the disease in Nigeria. Similar clinical signs have been reported elsewhere [26,33]; these authors also used the term hygroma for fluid accumulation in locations other than the joints. The hygromas are localized in carpal and other bursae and contain large numbers of the organisms [67,68]. The traditional Fulani cattle rearers practice ‘firing’ of the hygroma lesions, by using a hot knife to incise the swelling through the capsules, when large numbers of the localized brucellae are discharged from the hygroma and contaminate the environment, further encouraging the spread of the disease (see Additional file 1). The herd that had hygroma and was serologically negative is consistent with a previous report that 13% of brucellosis positive animals were serologically negative [69]. It is also possible that some of the hygromas observed in this herd may have been due to another aetiology, such as intermittent mild trauma to the precarpal area caused by lack of bedding or a poorly designed feed bunk [70]. This may partly explain the very high prevalence of hygromas seen amongst the seropositive animals in Adamawa state. Nevertheless, the presence of hygroma in one or more animals in a herd appeared to be a fairly specific predictor of herd seropositivity, with estimated specificity of 98.4%. It could therefore be used in participatory disease surveillance, although its estimated sensitivity of only 29.5% would mean that other signs of herd infection would also have to be considered.

Although not statistically significant, the prevalence of brucellosis was somewhat lower in *Bos taurus* breeds than amongst indigenous breeds. This finding is consistent with reports by Kubuafor *et al.*[25] in Ghana. Karimuribo *et al.*[29] in Tanzania stated that the proportion of seropositive animals was significantly higher in indigenous than in crossbred cattle. However, Muma *et al*. [42] reported no association between *Brucella* seropositivity and cattle breed. The better management in the exotic herds, stall or intensive feeding that minimizes contact between animals and herds may be responsible for the low prevalence. The distribution of breeds between management systems in this study varied, with highest number of Bunaji found in agro-pastoral followed by pastoral systems; Gudali in agro-pastoral and commercial systems; and *Bos taurus* were mainly in commercial farms. However, adjustment for management system did not change the result. Esuruoso [17] reported that the Ndama breed was the most affected breed in western Nigeria, while Cadmus *et al.*[18] found the Red Bororo breed to have the most positive reactors followed by Bunaji. Junaidu *et al*. [21] reported the Sokoto Gudali breed to have the highest prevalence, followed by Azuwarq, with Bunaji having the least prevalence. Genetic variation is an important factor in conferring resistance or tolerance of cattle breeds to a wide range of diseases, and the antibody response of animals classified as resistant to infection by *B. abortus* differed significantly from that of susceptible animals [71]. Significant genetic variability in resistance/susceptibility to brucellosis has been detected in cattle and associated with a 3’ untranslated polymorphism in the *Slc 1a1* gene [72]. This aspect needs further studies in Nigeria.

The prevalence of brucellosis was significantly higher in males than females, and this did not change after adjusting for age, management system, state or breed. This difference between sexes is consistent with reports by Chimana *et al.*[73] who recorded more seropositive cases in bulls (12.5%) compared to females (8.1%). However, our findings are contrary to other reports that showed significantly higher prevalence in females than males [20,21] or no difference between sexes [12,22,25]. Fifteen percent of the infected herds had animals with clinical evidence of orchitis and/or epididymitis (Table 1). Reports in Nigeria and elsewhere indicated that testes, epididymis and other accessory sex organs may be affected [5,6].

In the present study, the prevalence of brucellosis increased with age, with the odds of having brucellosis 3.8 times greater amongst cattle >7 years than those <4 years old. This is consistent with previous reports [21,25,74]. The higher prevalence of brucellosis in older cattle can be attributed to constant exposure of the cattle over time to the infectious agent. However, Cadmus *et al*. [18] observed no difference between cattle >3 years and 1–3 years old, whereas Matope *et al*. [31] reported decreased frequency of brucellosis with increasing age, with 2–4 years old having higher odds of being seropositive compared to those >7 years. They concluded that some older cows may not exhibit detectable antibody titres possibly due to latency, which is common in chronic brucellosis.

The significantly higher prevalence in non-pregnant compared to pregnant animals in this study did not change after adjusting for age, breed, state and management system. This finding is consistent with the observation in Ethiopia by Ibrahim *et al*. [64] but contrary to reports by Mekonnen *et al.*[75]. Pregnant cattle above five months of gestation are more susceptible to *Brucella* infection due to the preferential localization of *Brucella* in the uterus in which allantoic fluid factors such as erythritol stimulate the growth of *Brucella*[5]. However, the greater probability of abortion in infected animals could explain the higher seroprevalence in non-pregnant animals.

The difference in prevalence between non-lactating and lactating cows was not significant, consistent with reports by Mekonnen *et al.*[75] and Medeiros *et al*. [76] but inconsistent with findings by Ibrahim *et al*. [64] and Soomro [77]. A prevalence of 25% in lactating cows was recently reported in Nigeria by Junaidu *et al*. [21] and 80.7% in Pakistan by Soomro [77]. This is of public health importance particularly in those Fulanis observed to be drinking raw milk directly from the udder of the cow, since *B. abortus* has been isolated from raw and sour milk of Fulani cattle in Nigeria [19,62]. Brucellosis remains one of the most common zoonotic diseases worldwide with more than 500,000 human cases reported annually [78]; many of the farmers take no measures to protect themselves against brucellosis and are quite willing to drink unpasteurized milk. In this area, milk is usually preserved by souring, which does not destroy brucellae as they are preserved in the milk fat [62]. Unfortunately, infected farmers with symptoms of undulating fever and joint pain very rarely seek medical help, and if they do, the fever is usually ascribed to malaria or typhoid, therefore human brucellosis is likely to be greatly under-diagnosed.

## Conclusion

It is evident from this study that bovine brucellosis is endemic in northern Nigeria at a high prevalence, with the majority of herds in all management systems being infected and the traditional management systems, particularly of the pastoral Fulanis, and lack of control measures are encouraging the spread of the disease. Improved management systems are necessary. Further surveys in other locations, identification of resistant breeds, increased education and farmer extension, particularly amongst the nomadic Fulani, regarding the zoonotic risk associated with milk consumption and contact with aborted materials, and implementation of appropriate control measures are recommended. Food derived from animal sources must be properly cooked, isolation and slaughtering of seropositive reactors for brucellosis must be practiced and protective clothing must be provided for people dealing with infected cattle, blood and meat. Integrating vaccination against brucellosis into the annual vaccination programme of livestock is highly recommended. Cooperation between neighbouring countries and intensifying border patrols in order to restrict movement of cattle across borders are also suggested.

## Abbreviations

c-ELISA: Competitive Enzyme-linked Immunosorbent Assay; RBPT: Rose-Bengal Plate-agglutination Test; SAT: Serum Agglutination Test; CI: Confidence Interval; FP: False Positive.

## Competing interests

The authors declare that they have no competing interests.

## Authors’ contributions

HMM is a PhD student who conceptualized and designed the study, collected the samples and questionnaires from the field, did the RBPT and c-ELISA testing of the samples with the help of the laboratory assistants, performed the statistical analysis and interpretation, and drafted the manuscript. PNT was the promoter and project leader, he was involved in the design of the study and statistical analysis and interpretation of the data provided, and revised the manuscript critically. JK was also involved in the design, analysis and interpretation of data and revised the manuscript. PCI was the co-promoter, who also participated in the design of the project and helped in revision of the manuscript. All the authors read and approved the final manuscript.

## Authors’ information

HMM has a DVM, MSc in Theriogenology and pursuing a PhD in the Department of Production Animal Studies, Faculty of Veterinary Science, University of Pretoria. PNT has a BVSc, MMedVet and PhD and is Associate Professor of Veterinary Epidemiology in the Department of Production Animal Studies, Faculty of Veterinary Science, University of Pretoria. JK has a DVM, MSc and PhD and is an Associate Professor in Veterinary Public Health. PCI has a BVSc, MMedVet and PhD and is Professor in Theriogenology. He is presently the Head of the Department of Production Animal Studies, Faculty of Veterinary Science, University of Pretoria.

## Supplementary Material

Additional file 1: ** Different hygroma lesions encountered, with capsules at *post mortem* and firing of the hygroma lesions practiced by the pastoral Fulanis.** Click here for file
