# Supplementary material for: A large seroprevalence survey of brucellosis in cattle herds under diverse production systems in northern Nigeria
Source: BMC Vet Res. 2012 Aug 25;8:144. doi: 10.1186/1746-6148-8-144 (PMC3482151; doi:10.1186/1746-6148-8-144)
Supplement: Additional file 1: — Different hygroma lesions encountered, with capsules at post mortem and firing of the hygroma lesions practiced by the pastoral Fulanis. [file 1746-6148-8-144-S1.doc]

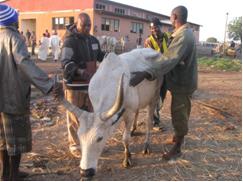

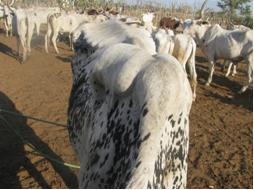


Fig. 3a. Hygroma on right cervical region Fig. 3b. Hygroma on withers/thoracic spine


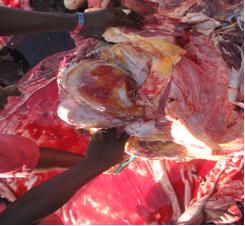

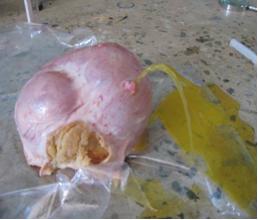


Fig. 3c. Capsules from hygroma at *post mortem* Fig 3d. Capsule punctured with fluid at *post mortem*


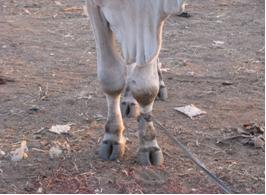

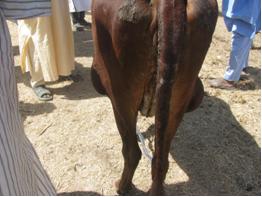


Fig 3e. Bilateral carpal hygroma Fig 3f. Bilateral hygroma of stifle joint


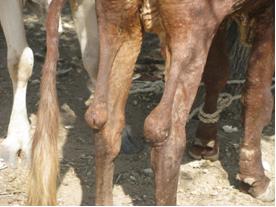

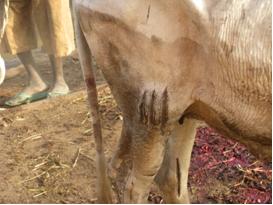


Fig 3g. Bilateral hygroma of hock joint Fig. 3h. Firing of hygroma by pastoral Fulanis

Additional file 1. Different hygroma lesions encountered, with capsules at *post mortem* and firing of the hygroma lesions practiced by the pastoral Fulanis
